# Outcomes of Influenza A(H1N1)pdm09 Virus Infection: Results from Two International Cohort Studies

**DOI:** 10.1371/journal.pone.0101785

**Published:** 2014-07-08

**Authors:** Ruth Lynfield, Richard Davey, Dominic E. Dwyer, Marcelo H. Losso, Deborah Wentworth, Alessandro Cozzi-Lepri, Kathy Herman-Lamin, Grazyna Cholewinska, Daniel David, Stefan Kuetter, Zelalem Ternesgen, Timothy M. Uyeki, H. Clifford Lane, Jens Lundgren, James D. Neaton, for the INSIGHT Influenza Study Group

**Affiliations:** 1 Infectious Disease Division, Minnesota Department of Health, St. Paul, Minnesota, United States of America; 2 National Institute of Allergy and Infectious Diseases, National Institutes of Health, Bethesda, Maryland, United States of America; 3 Department of Virology, Centre for Infectious Diseases and Microbiology, Westmead Hospital and University of Sydney, Westmead, New South Wales, Australia; 4 HIV Unit, Department of Medicine, Hospital José María Ramos Mejía, Buenos Aires, Argentina; 5 Division of Biostatistics, University of Minnesota, Minneapolis, Minnesota, United States of America; 6 Research Department of Infection and Population Health, University College London, London, England, United Kingdom; 7 Hospital for Infectious Diseases, Warsaw, Poland; 8 Hospital Rawson, Infectología, Cordoba, Argentina; 9 Marlow Medical Group, Marlow, United Kingdom; 10 Mayo Clinic, Rochester, Minnesota, United States of America; 11 Influenza Division, Centers for Disease Control and Prevention, Atlanta, Georgia, United States of America; 12 Department of Infectious Diseases, Copenhagen University Hospital/Rigshospitalet & University of Copenhagen, Copenhagen, Denmark; University of Liverpool, United Kingdom

## Abstract

**Background:**

Data from prospectively planned cohort studies on risk of major clinical outcomes and prognostic factors for patients with influenza A(H1N1)pdm09 virus are limited. In 2009, in order to assess outcomes and evaluate risk factors for progression of illness, two cohort studies were initiated: FLU 002 in outpatients and FLU 003 in hospitalized patients.

**Methods and Findings:**

Between October 2009 and December 2012, adults with influenza-like illness (ILI) were enrolled; outpatients were followed for 14 days and inpatients for 60 days. Disease progression was defined as hospitalization and/or death for outpatients, and hospitalization for >28 days, transfer to intensive care unit (ICU) if enrolled from general ward, and/or death for inpatients. Infection was confirmed by RT-PCR. 590 FLU 002 and 392 FLU 003 patients with influenza A (H1N1)pdm09 were enrolled from 81 sites in 17 countries at 2 days (IQR 1–3) and 6 days (IQR 4–10) following ILI onset, respectively. Disease progression was experienced by 29 (1 death) outpatients (5.1%; 95% CI: 3.4–7.2%) and 80 inpatients [death (32), hospitalization >28 days (43) or ICU transfer (20)] (21.6%; 95% CI: 17.5–26.2%). Disease progression (death) for hospitalized patients was 53.1% (26.6%) and 12.8% (3.8%), respectively, for those enrolled in the ICU and general ward. In pooled analyses for both studies, predictors of disease progression were age, longer duration of symptoms at enrollment and immunosuppression. Patients hospitalized during the pandemic period had a poorer prognosis than in subsequent seasons.

**Conclusions:**

Patients with influenza A(H1N1)pdm09, particularly when requiring hospital admission, are at high risk for disease progression, especially if they are older, immunodeficient, or admitted late in infection. These data reinforce the need for international trials of novel treatment strategies for influenza infection and serve as a reminder of the need to monitor the severity of seasonal and pandemic influenza epidemics globally.

**Trial Registration:**

ClinicalTrials.gov Identifiers: FLU 002- NCT01056354, FLU 003- NCT01056185.

## Introduction

The emergence of influenza A(H1N1)pdm09 virus in 2009 highlighted the importance of having infrastructures in place to conduct research that would inform patient management on emerging viruses [1]. Although surveillance systems for influenza exist in many parts of the world, these systems tend to be either laboratory-based, focused on characterizing circulating virus strains for vaccine strain selection or antiviral resistance monitoring, or include clinical data on outpatients or hospitalized patients, but do not include follow-up [2]–[6].

Follow-up studies of patients diagnosed with influenza are necessary to estimate the percentage that progress to death or respiratory failure, or who require prolonged hospitalization. Clinical data close to the time of diagnosis are needed to study risk factors for progression. Ideally, such data would be available from geographically diverse settings over several influenza seasons with different influenza viruses in order to understand changing patterns of disease and risk factors of progression. These data could inform clinical management strategies as well as the design of intervention studies.

In response to the urgent need for such follow-up data, in 2009 the National Institutes of Health funded two international cohort studies of patients with A(H1N1)pdm09 virus infection. In this report, we describe outcomes of outpatients and hospitalized patients with influenza A(H1N1)pdm09 virus infection and examine risk factors for progression of their illness. To our knowledge, other global cohort data which include a follow-up period, from geographically diverse settings for patients with a broad range of severity of illness at the time enrollment do not exist.

## Methods

The International Network for Strategic Initiatives in Global HIV Trials (INSIGHT) rapidly initiated two international cohort studies of patients with A(H1N1)pdm09 virus infection in 2009. Although originally designed to conduct large HIV treatment trials, INSIGHT adapted and expanded its global network to include the study of influenza. One study (FLU 002) enrolled patients seeking assessment for influenza-like illness (ILI) as outpatients; a second study (FLU 003) enrolled patients who had been hospitalized for complications associated with influenza. The study designs of both studies have been described elsewhere [7].

Briefly, the two studies were designed to cover a broad clinical spectrum of A(H1N1)pdm09 virus infection in adults (≥18 years of age), ranging from outpatients presenting with mild ILI symptoms (FLU 002) to those with more serious disease requiring hospitalization (FLU 003), and both studies included follow-up periods. Initially, sites were not open to enrollment until A(H1N1)pdm09 virus was circulating in their geographic areas. Later these studies were expanded to include other seasonal influenza viruses; outcomes for patients with other influenza viruses will be included in a subsequent report.

For both studies, information collected at the time of enrollment included patient demographics, height, weight and vital signs; date of ILI onset; medical history, including underlying conditions, pregnancy status, and smoking history, and use of neuraminidase inhibitors to prevent or treat influenza. For FLU 003, the type of complication prompting hospital admission was also collected.

### Ethics Statement

The FLU 002 and FLU 003 protocols were approved by the institutional review boards (IRB) or institutional ethics committees (IEC) at the University of Minnesota and at each of the participating clinical sites worldwide (see Appendix S1). Written documentation of IRB/IEC approval to each site Principal Investigator was a required element in the site registration process that preceded site activation as a study center. Copies of these approval letters are filed with the central coordinating center at the University of Minnesota. All patients (or proxy) gave signed informed consent prior to enrollment.

### Disease Progression Outcomes

Enrolled outpatients with ILI were followed for 14 days for hospitalization or death. Henceforth for FLU 002 patients, this composite outcome is referred to as “disease progression”. At 14 days the resolution of symptoms was also assessed.

Enrolled hospitalized patients were followed for 60 days. For general ward patients, outcomes assessed included death, ICU admission and/or mechanical ventilation, or prolonged hospitalization; the latter was defined as an inpatient stay exceeding 28 days of the 60-day follow-up period, not necessarily consecutively. For patients enrolled after ICU admission, death or prolonged hospitalization for >28 days were the primary outcomes. For FLU 003 patients, this composite outcome, stratified according to whether patients were enrolled from a general ward or ICU, is referred to as “disease progression”. Length of hospitalization, resolution of symptoms, and resumption of normal activities were assessed at 28 and 60 days after enrollment.

### Methods for the Laboratory Diagnosis of A(H1N1)pdm09 virus infection

In both studies, respiratory (nasal and oropharyngeal) swabs were collected at enrollment for influenza testing. The combined respiratory sample was sent to one of two central laboratories for influenza testing (SAIC Frederick, Inc, Maryland or Advanced BioMedical Laboratories, New Jersey) by reverse transcription polymerase chain reaction (RT-PCR) assay using specific primers and probes for detection of influenza A, (seasonal H1, H1N1pdm09, H3), and B viruses. In FLU 003, a local RT-PCR test result was required either prior to enrollment (for confirmed diagnoses) or at the time of enrollment (for suspected diagnoses). Initially, local RT-PCR test results were only recorded as influenza A positive or negative; after the first year, influenza A subtyping results were recorded. We assessed the discordance of local and central RT-PCR results. Results are shown in Appendix S2 with a rationale for inclusion of patients in each A(H1N1)pdm09 virus-infected cohort.

### Definition of A(H1N1)pdm09 Virus-Infected Cohorts Based on RT-PCR Results

Outpatients enrolled with A(H1N1)pdm09 confirmed at the central laboratories are included in the FLU 002 cohort. The FLU 003 hospitalized cohort includes patients with A(H1N1)pdm09 virus infection confirmed at a central laboratory and patients who tested positive for influenza A by a local laboratory and negative for influenza A at a central laboratory during the initial 6 months of enrollment when A(H1N1)pdm09 virus was highly prevalent and the results of local RT-PCR testing did not record the influenza A subtype (see Appendix S2).

### Co-Pathogen Substudy

In a random subsample of 333 patients with A(H1N1)pdm09 virus infection, a tandem multiplex PCR (AusDiagnostics, Sydney Australia) was performed on upper respiratory specimens to estimate the prevalence of potential co-pathogens in each study [8]. These laboratory analyses were performed at the Centre for Infectious Diseases and Microbiology Laboratory Services, Westmead Hospital, Westmead, New South Wales, Australia.

### Statistical Analyses

Descriptive statistics were used to describe the characteristics of patients enrolled in the two cohort studies. Cross-sectional comparisons of patients in the two studies were performed to assess factors potentially contributing to disease severity: odds ratios (ORs) (hospitalized patients versus outpatients) and 95% confidence intervals (CIs) are cited. Unadjusted (univariable) and adjusted (multivariable) ORs are cited. Similar analyses were done for the subsample of patients for whom tandem multiplex PCR for other pathogens was performed.

The percentage of patients developing disease progression during follow-up was computed for each study. In addition, cumulative mortality for patients in FLU 003 is summarized with Kaplan-Meier plots. For these analyses, follow-up was censored at the end of follow-up (60 days) or the date of last contact (e.g., discharge or day 28) for those who did not complete the full follow-up. Logistic regression was used to study baseline predictors of disease progression and mortality. Prognostic factors for disease progression were determined separately for the two studies and for pooled data from the two studies. Unadjusted and adjusted ORs are cited along with 95% CIs and p-values. In expanded models, an interaction term (covariate x study) was included in the logistic model to assess whether associations with disease progression differed for FLU 002 and FLU 003.

Height and weight data, used to determine body mass index (BMI), were available for 91.0% of those enrolled. Date of onset of symptoms for ILI and smoking prevalence data were available for 98.7% and 99.2% of enrolled patients respectively. Other baseline covariate data were present for all patients. To minimize bias and increase power for multiple regression analyses that require complete covariate information for each patient, multiple imputation was used to predict values that were substituted for the missing data. The imputation was done in an iterative manner using the baseline covariate data available. The regression coefficients from five rounds of imputation were used to obtain the ORs. The imputation had little effect on the univariable analyses, therefore summary statistics from these analyses are based on the observed data. In a sensitivity analysis, a complete case analysis was performed and adjusted ORs were estimated for all of the baseline variables excluding BMI. Estimates similar to those based on multiple imputation were obtained (data not shown).

All statistical tests are two-tailed and p-values less than 0.05 were considered to indicate statistical significance. Statistical analyses were performed using SAS (Version 9.3).

## Results

**Figure 1:**
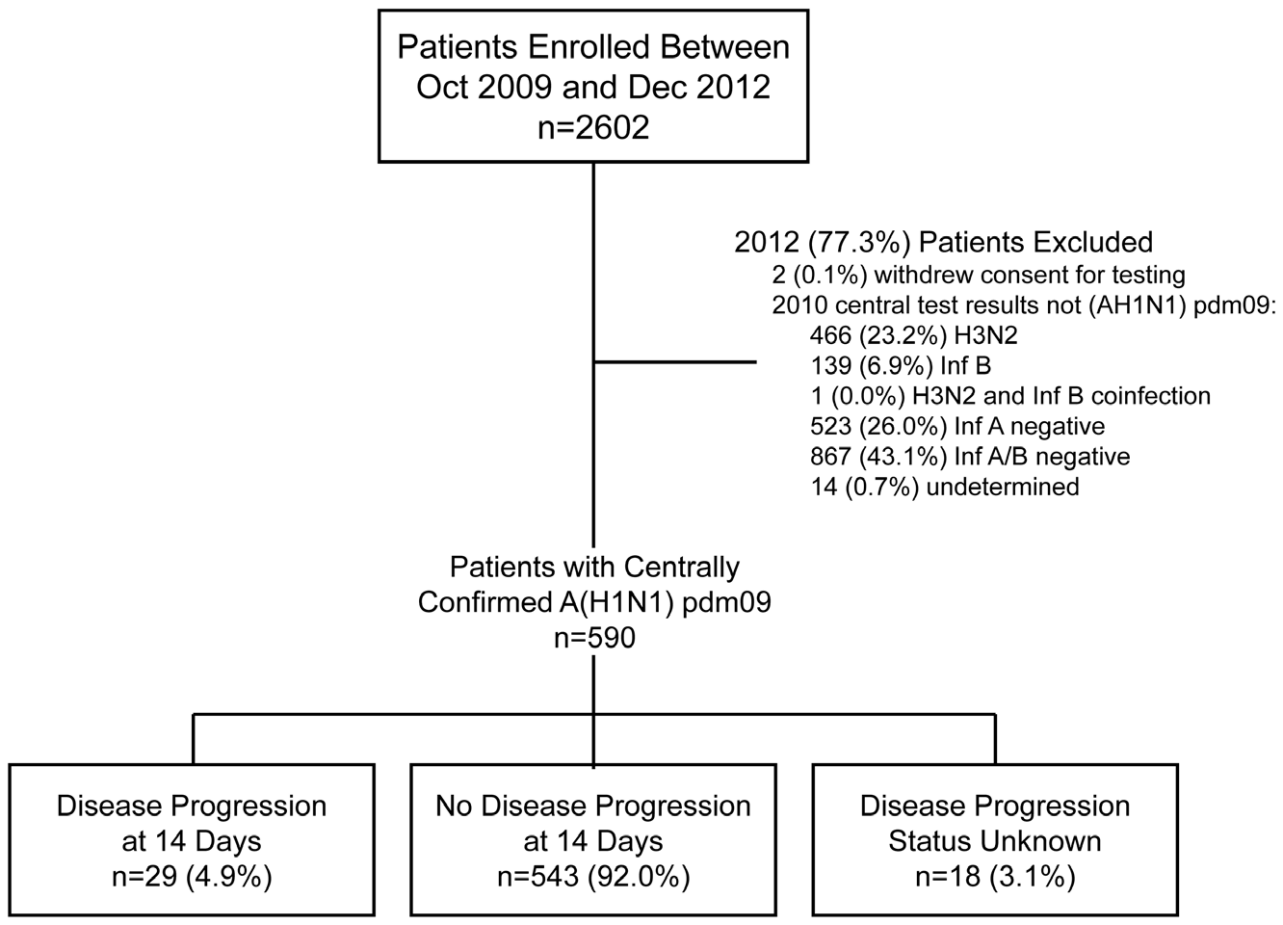
FLU 002 flow diagram.

Between October 2009 and December 2012, 2,602 patients were enrolled as outpatients in FLU 002, among whom 590 (23%) had laboratory-confirmed A(H1N1)pdm09 virus infection (Figure 1). Most (75%) patients with A(H1N1)pdm09 virus infection in FLU 002 were enrolled between October 2009 and September 2010 (Table 1) due to the declining prevalence of A(H1N1)pdm09 virus after 2010. During October 2009 through September 2010, 442 (94%) of 469 patients with a RT-PCR diagnosis of influenza at a central laboratory had A(H1N1)pdm09 virus infection (data not shown). The prevalence of A(H1N1)pdm09 virus over the next two years was 29% (119 of 410 patients) for patients enrolled between October 2010 and September 2011 and 9% (29 of 316 patients) for those enrolled between October 2011 and December 2012. After September 2010, A (H3N2) virus became the predominant influenza virus identified (data not shown).

**Table 1:** Baseline characteristics of A(H1N1)pdm09-infected participants enrolled in FLU002.

Season of enrollment	Oct 2009-Sep 2010	442 (74.9%)
	Oct 2010-Sep 2011	119 (20.2%)
	Oct 2011-Dec 2012	29 (4.9%)
Age - median (IQR)	All patients	30 (24, 42)
	Oct 2009-Sep 2010 enrollment	29 (23, 39)
	Oct 2010-Dec 2012 enrollment	35 (28, 47)
Gender	Female - no. (%)	307 (52.0%)
Race/ethnicity	Asian - no. (%)	172 (29.2%)
	Black - no. (%)	34 (5.8%)
	White/other - no. (%)	390 (66.1%)
Influenza vaccine**	All patients	82 (14.0%)
	Oct 2009-Sep 2010 enrollment	63 (14.3%)
	Oct 2010-Dec 2012 enrollment	19 (13.0%)
Other baseline characteristics	BMI - median (IQR)	23.7 (21.3, 27.5)
	BMI≥40 kg/m^2^ - no. (%)	10 (1.9%)
	Smoker - no. (%)	121 (20.6%)
	Pregnant * - no. (%)	5 (2.0%)
	Days since symptom onset - median (IQR)	2 (1, 3)
Medical history	Antivirals in past 14 days - no. (%)	15 (2.5%)
	Asthma/COPD - no. (%)	40 (6.8%)
	Diabetes - no. (%)	12 (2.0%)
	CVD/liver/renal disease - no. (%)	13 (2.2%)
	HIV/other immune dysfunction - no. (%)	55 (9.3%)

*Currently or within previous 2 weeks, percent of women ≤45 years.

**Receipt of influenza vaccine during current season.

**Figure 2:**
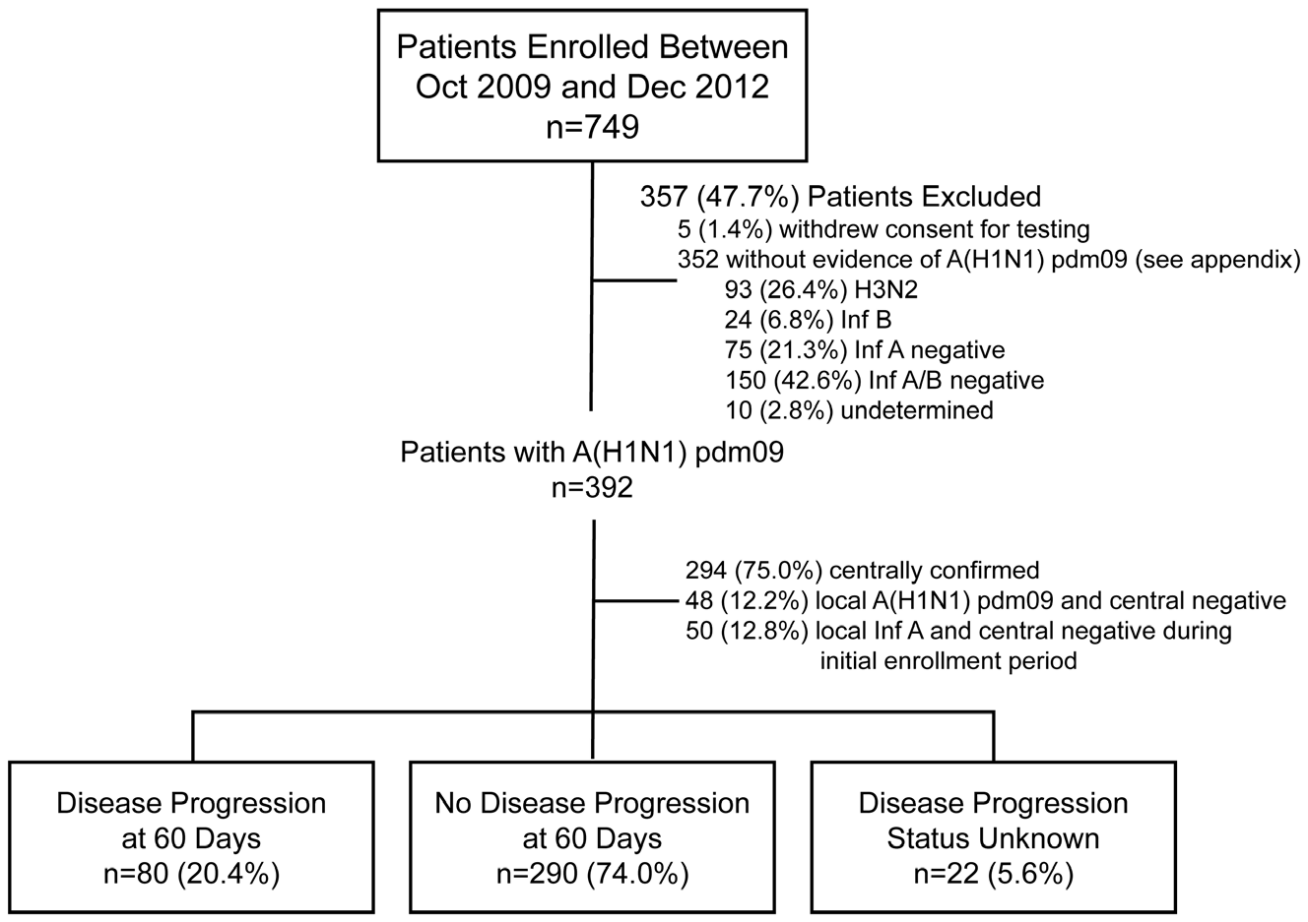
FLU 003 flow diagram.

In FLU 003, 749 hospitalized patients were enrolled and 392 (52%) had laboratory-confirmed A(H1N1)pdm09 virus infection. In both FLU 002 and FLU 003, most of the patients excluded from this analysis had tested negative for influenza A and/or B (Figures 1 and 2).

### Baseline Characteristics of Patients with A(H1N1)pdm09 Virus Infection Enrolled in FLU 002

In FLU 002, outpatients with A(H1N1)pdm09 virus infection were enrolled by 53 sites in 15 countries (see Acknowledgements for number enrolled by country). Asian sites enrolled 20.3% of patients; 4.1% of patients were from Australia; 46.1% from Europe; 8.0% from South America; and 21.5% from the United States. The median age of enrolled outpatients with A(H1N1)pdm09 virus infection was 30 years; those enrolled in the first year (2009–2010) had a median age that was 6 years younger than in subsequent periods (29 versus 35 years; p<0.001 for difference) (Table 1). Fifty-two percent of patients were female; 1.9% had a BMI of ≥40 kg/m^2^; 21% reported smoking; and 2% of the women aged ≤45 years of age were pregnant at the time of enrollment or within the previous two weeks. Median time from the onset of symptoms to enrollment was two days; for 75% of patients this time was three days or less. Fifty-five patients (9.3%) had HIV infection or other immune dysfunction; 50 of the 55 patients had HIV infection, reflecting the fact that many of the infectious disease clinics participating in FLU 002 cared for patients with HIV infection. Fifteen (2.5%) patients were prescribed influenza antivirals (all oseltamivir) in the 14 days prior to enrollment. On the day of enrollment, 28% of patients were prescribed antiviral treatment (data not shown).

### Disease Progression and Other Outcomes for Patients with A(H1N1)pdm09 Virus Infection Enrolled in FLU 002

Disease progression status at day 14 was available for 572 (96.9%) of enrolled patients in FLU 002. Twenty-nine patients (5.1%; 95% CI: 3.4–7.2%) experienced disease progression during the 14-day follow-up period; 28 (4.9%) required hospitalization and one patient died (Table 2). Of the 28 patients initially enrolled as outpatients who were subsequently hospitalized, 12 (42.9%) were admitted to the hospital later on the same day as study enrollment.

**Table 2:** Outcomes through 14 days of follow-up for A(H1N1)pdm09-infected patients enrolled in FLU002.

	No.	Pct.	95% CI
Death	1	0.17	0.0–1.0
Hospitalized during follow-up	28	4.9	3.3–7.0
Death or hospitalization (disease progression)	29	5.1	3.4–7.2
Death, hospitalization, or influenza symptoms	127	22.2	18.9–25.8

One hundred and five outpatients (18.3%; 95% CI 15.2 to 21.7%) with A(H1N1)pdm09 virus infection reported that their symptoms had not resolved by day 14; the percentage who died, were hospitalized, or continued to report symptoms at day 14 was 22.2% (95% CI: 18.9 to 25.8%).

### Baseline Characteristics of Patients with A(H1N1)pdm09 Virus Infection Enrolled in FLU 003

In FLU 003, hospitalized patients with A(H1N1)pdm09 virus infection were enrolled at 56 sites in 16 countries; sites in 15 of these countries also enrolled patients in FLU 002 (enrollment by country is given in Acknowledgments). Asian sites enrolled 7.1% of patients; 10.5% of patients were from Australia; 70.4% from Europe; 2.0% from South America; and 10.0% from the United States. Fifty-five percent were enrolled between October 2009 and September 2010 (Table 3). Three hundred and seven (78.3%) of the 392 A(H1N1)pdm09 patients were enrolled from a general hospital ward and 85 (21.7%) were enrolled from an ICU. The median age of hospitalized patients with A(H1N1)pdm09 was 48 years; those enrolled in the first calendar year of enrollment had a median age that was seven years younger (44 versus 51 years; p = 0.001 for difference) than in subsequent years. This age difference was evident both for patients enrolled from the general ward and from the ICU. Fifty-one percent of patients were female; 11% were Asian, 4% were black, and 85% were white/other; the median BMI was 26 kg/m^2^; 5.3% had a BMI of ≥40 kg/m^2^; 30% reported smoking; and 25% of the women aged ≤45 years were pregnant. Fifty-three patients (13.5%) had HIV infection or other immune dysfunction; 14 of the 53 patients had HIV infection. Median time from the onset of symptoms to enrollment was five days for patients enrolled in the general ward and 10 days for patients enrolled from an ICU. Eighteen patients (4.7%) developed ILI symptoms after being hospitalized for some other condition; the median (IQR) time between admission and ILI symptom onset was 8 days (IQR: 5–18). Excluding the patients who likely acquired A(H1N1)pdm09 virus infection in the hospital, the median time from admission to enrollment was two days for patients enrolled from a general ward and 5 days for patients enrolled while in an ICU.

**Table 3:** Baseline characteristics of A(H1N1)pdm09-infected participants enrolled in FLU003.

		FLU 003 Ward	FLU 003 ICU	Total
		N = 307	N = 85	N = 392
Season of enrollment	Oct 2009-Sep 2010	165 (53.7)	52 (61.2)	217 (55.4)
	Oct 2010-Sep 2011	132 (43.0)	31 (36.5)	163 (41.6)
	Oct 2011-Dec 2012	10 (3.3)	2 (2.4)	12 (3.1)
Age - median (IQR)	All patients	48 (36, 60)	46 (31, 56)	48 (35, 59)
	Oct 2009-Sep 2010	44 (34, 56)	40 (28, 57)	44 (32, 56)
	Oct 2010-Dec 2012	51 (36, 62)	48 (40, 56)	51 (38, 62)
Gender	Female - no. (%)	163 (53.1)	37 (43.5)	200 (51.0)
Race/ethnicity	Asian - no. (%)	28 (9.1)	14 (16.5)	42 (10.7)
	Black - no. (%)	14 (4.6)	3 (3.5)	17 (4.3)
	White/other - no. (%)	265 (86.3)	68 (80.0)	333 (84.9)
Other baseline characteristics	BMI - median (IQR)	25.6 (22.9, 30.0)	27.3 (24.8, 31.7)	26.0 (23.1, 30.4)
	Smoker - no. (%)	97 (31.7)	19 (23.5)	116 (30.0)
	Pregnant * - no. (%)	15 (18.3)	11 (50.0)	26 (25.0)
	Days since symptom onset - median (IQR)	5 (3, 8)	10 (6, 14)	6 (4, 10)
	Antiviral drugs in previous 14 days - no. (%)	192 (62.5)	66 (77.6)	258 (65.8)
Influenza vaccine**	All patients	70 (23.8)	9 (13.6)	79 (21.9)
	Oct 2009-Sep 2010	36 (22.5)	4 (11.1)	40 (20.4)
	Oct 2010-Dec 2012	34 (25.4)	5 (16.7)	39 (23.8)
Medical History	Asthma/COPD - no. (%)	91 (29.6)	13 (15.3)	104 (26.5)
	Diabetes - no. (%)	27 (8.8)	12 (14.1)	39 (9.9)
	CVD/liver/renal disease - no. (%)	61 (19.9)	15 (17.6)	76 (19.4)
	HIV/other immune dysfunction - no. (%)	43 (14.0)	10 (11.8)	53 (13.5)
Complications Defining Eligibility	Supplemental oxygen required - no. (%)	239 (77.9)	81 (95.3)	320 (81.6)
	Exacerbation of comorbidity - no. (%)	120 (39.1)	18 (21.2)	138 (35.2)
	Vasopressors required - no. (%)	10 (3.3)	28 (32.9)	38 (9.7)
	Acute renal failure - no. (%)	14 (4.6)	19 (22.4)	33 (8.4)
	Acute liver failure - no. (%)	6 (2.0)	2 (2.4)	8 (2.0)
	Pregnancy complications - no. (%)	5 (1.6)	5 (5.9)	10 (2.6)
	Other organ dysfunction - no. (%)	15 (4.9)	7 (8.2)	22 (5.6)
Other Complications	Bacterial pneumoniae - no. (%)	83 (27.0)	32 (37.6)	115 (29.3)
	Dehydration requiring IV - no. (%)	92 (30.0)	34 (40.0)	126 (32.1)
	Enteritis - no. (%)	13 (4.2)	8 (9.4)	21 (5.4)
	Septicemia - no. (%)	7 (2.3)	8 (9.4)	15 (3.8)

*Currently or within previous 2 weeks, percent of women ≤45 years.

**Receipt of influenza vaccine during current season.

As would be expected, by most measures of disease severity assessed (medical history, complications defining eligibility, and other complications) patients enrolled in the ICU had more severe illness than those enrolled from the general ward. Exceptions were a history of asthma/chronic obstructive pulmonary disease (COPD), cardiovascular disease (CVD), liver or renal disease, and exacerbations of other co-morbidities which were more common among patients enrolled from a general ward than those enrolled from an ICU.

Two hundred and fifty-eight patients (65.8%) reported taking antivirals for influenza in the 14 days prior to enrollment; 256 were taking oseltamivir and 5 were taking zanamivir (3 following a course of oseltamivir). For patients taking an antiviral before enrollment, 46.6% reported starting antiviral treatment within 3 days of the onset of ILI symptoms; the median time between symptom onset and starting antiviral treatment was four days (IQR: 2–7).

### Disease Progression and Other Outcomes for Patients with A(H1N1)pdm09 Virus Infection Enrolled in FLU 003

Disease progression status was known at day 60 for 370 (94.4%) patients enrolled in FLU 003 (Figure 2). During the 60-day follow-up period, 80 (21.6%; 95% CI: 17.5 to 26.2%) patients developed disease progression; for those enrolled in the general ward and ICU, 37 (12.8%; 95% CI: 9.2 to 17.2%) and 43 (53.1%; 95% CI: 41.7 to 64.3%) patients experienced disease progression, respectively (Table 4).

**Table 4:** Major outcomes through 60 days of follow-up for A(H1N1)pdm09-infected patients enrolled in FLU003.

	Enrolled from			Enrolled					
	General Ward			From ICU			Total		
	No.	Pct.	CI	No.	Pct.	CI	No.	Pct.	CI
Death	11	3.8	1.9–6.8	21	26.6	17.3–37.7	32	8.7	6.1–12.1
Hospitalization >28 days	17	5.6	3.3–8.8	26	31.0	21.3–42.0	43	11.1	8.1–14.6
(from enrollment)									
Progressed to ICU,	20	6.5	4.0–9.9						
ECMO or intubation									
Any of above	37	12.8	9.2–17.2	43	53.1	41.7–64.3	80	21.6	17.5–26.2
(combination endpoint)									

**Figure 3:**
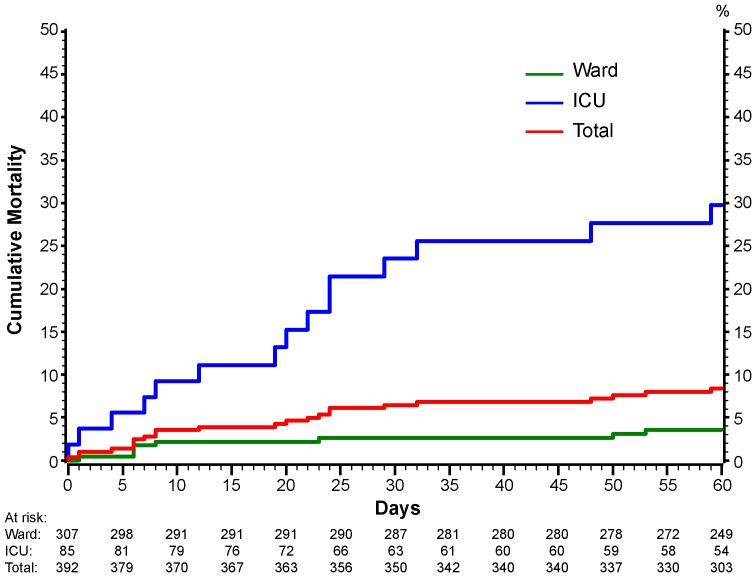
Cumulative percentage of patients with death from any cause in FLU 003 according to location of enrollment. The number of patients at risk at each timepoint are given below the graph.

Thirty-two patients (8.7%; 95% CI: 6.1 to 12.1%) died during the 60-day follow-up period. Twenty seven of these 32 patients died before discharge from the hospital at which they were enrolled. Figure 3 shows Kaplan-Meier plots for all-cause mortality for those enrolled in the general ward and the ICU. Cumulative mortality at 14, 28 and 60 days for those enrolled from a general ward were 2.3, 2.7, and 3.7%; for those enrolled from an ICU, these percentages were 9.4, 19.2, and 25.6%, respectively (95% CIs are given in the legend of Figure 3).

The number of days hospitalized since the time of enrollment, taking into account re-admissions (49 patients had at least one re-admission), was 5 days (IQR 2–12); for general ward patients the median number was 4 days (IQR 1–8) and for those enrolled from the ICU the median number was 15 days (IQR 8–32). For the estimation of these medians, deaths were assigned a worst-case time of 60 days.

At 28 days of follow-up among 289 surviving patients who had been discharged and attended the follow-up visit, 25.3% (95% CI: 20.3 to 30.7%) indicated that influenza symptoms had not resolved; 38.5% (95% CI; 30.3 to 46.7%) of patients had not resumed normal activities. At 60 days of follow-up among 292 surviving patients who had been discharged and attended the follow-up visit, 14.7% of patients (95% CI: 10.7 to 19.3%) indicated that symptoms had not resolved; 24.3% (95% CI: 17.4 to 32.2%) indicated that they had not resumed normal activities.

### Comparison of Baseline Characteristics for FLU 002 and FLU 003 Patients with A(H1N1)pdm09 Virus Infection

Table 5 summarizes the differences between FLU 002 and FLU 003 patients. In multivariable analyses, compared to outpatients, hospitalized patients were older, more likely to be female, have a history of asthma or COPD, and a history of CVD, liver or renal disease, and based on linear trend, have greater BMI and a longer duration of symptoms (p<0.05 for all). In addition, in the first year significantly fewer hospitalized patients were enrolled.

**Table 5:** Baseline characteristics associated with disease severity at entry: FLU002 versus FLU003.

				Unadjusted		Adjusted		
Characteristic		FLU 002	FLU 003	OR*	p-value	OR**	*95% C.I.*	p-value
Race	Asian - %	29.2	10.7	0.28	<.001	0.73	*0.44, 1.22*	.23
	Black - %	5.8	4.3	0.58	.07	0.41	*0.17, 1.03*	.06
	White/other - %	66.1	84.9	ref		ref		
Other demographics	Age - median (IQR)	30 (24, 42)	48 (35, 59)	1.95	<.001	1.48	*1.28, 1.70*	<.001
	Female - %	52.0	51.0	0.96	.76	1.48	*1.01, 2.17*	.04
BMI (kg/m^2^)***	BMI <30 - %	83.7	73.5	ref		ref		
	BMI 30–39.9 - %	14.4	21.2	1.67	.004	1.78	*1.11, 2.85*	.02
	BMI ≥40 - %	1.9	5.3	3.22	.003	2.84	*0.94, 8.60*	.07
Onset to enrollment****	0–3 days - %	81.3	22.4	ref		ref		
	4–5 days - %	12.2	23.2	6.89	<.001	5.91	*3.77, 9.27*	<.001
	6+ days - %	6.5	54.5	30.7	<.001	25.7	*16.2, 41.0*	<.001
Season of enrollment	Oct 2009-Sep 2010 - %	74.9	55.4	0.42	<.001	0.62	*0.42, 0.92*	.02
	Oct 2010-Dec 2012 - %	25.1	44.6	ref		ref		
Medical History	Smoker - %	20.6	30.0	1.65	<.001	1.35	*0.88, 2.07*	.17
	Asthma/COPD - %	6.8	26.5	4.96	<.001	3.49	*2.05, 5.93*	<.001
	Diabetes - %	2.0	9.9	5.32	<.001	1.71	*0.67, 4.37*	.27
	CVD/liver/renal - %	2.2	19.4	10.7	<.001	7.52	*3.46, 16.3*	<.001
	HIV/other immune - %	9.3	13.5	1.52	.04	0.74	*0.41, 1.35*	.32
Co-infections*****	Non-influenza virus - %	14.9	15.3	1.03	.92	1.22	*0.49, 3.03*	.66
	S. aureus - %	30.6	19.4	0.54	.04	0.52	*0.24, 1.14*	.10
	S. pneumoniae - %	22.6	16.3	0.67	.20	0.64	*0.29, 1.41*	.26
	M. pneumoniae - %	23.0	25.5	1.15	.62	1.76	*0.81, 3.81*	.15
	Bordetella - %	6.8	11.2	1.73	.18	1.53	*0.55, 4.23*	.41

*Odds ratio for FLU003 vs. FLU002. OR for age is for 10 years older.

**Multivariate model using imputed data with adjustment for all variables listed except coinfections.

***P-value for linear trend from multivariate model = .0010

****P-value for linear trend from multivariate model = .0000

*****As above for a subset of patients (n = 333) analyzed for co-infections.

We also assessed whether pregnant women were more likely to be enrolled in FLU 003 than FLU 002. Among women aged ≤45 years, there were more pregnant women in FLU 003 than in FLU 002 (see Tables 1 and 2) (univariable OR = 16.0; 95% CI: 5.9 to 43.1). After covariate adjustment, this OR was 32.5 (95% CI: 8.9 to 118.6).

**Figure 4:**
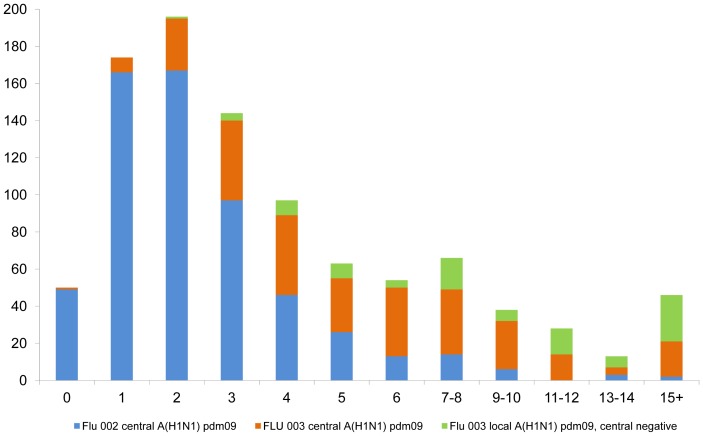
Frequency distribution of number of days between onset of ILI symptoms and enrollment for patients in FLU 002 and FLU 003.

Figure 4 gives the frequency distribution of the number of days between the development of A(H1N1)pdm09-related symptoms and enrollment for patients in FLU 002 and FLU 003. This graphical depiction illustrates the longer period of time between symptom onset and enrollment for patients in FLU 003. Also, for those in FLU 003 for whom central laboratory RT-PCR results were negative, but with positive results for A(H1N1)pdm09 virus infection by a local laboratory, this time was even longer than for those with centrally confirmed A(H1N1)pdm09 virus infection in FLU 003(median time between illness onset and enrollment for these patients was 10 days; IQR: 6–15). Overall, there was a median of 2 (IQR: 1–4) days between local and central swab collection (see Appendix S2).

The prevalence of other co-pathogens was compared for a subsample of respiratory specimens for 235 patients in FLU 002 and 98 patients in FLU 003 (bottom of Table 5). With the exception of *S. aureus*, which was more common in FLU 002 than FLU 003 in univariable analysis but not in multivariable analyses, the prevalence of potential co-pathogens in the upper respiratory tract did not differ significantly between patients in the two studies.

### Relationship of Baseline Factors with Disease Progression for Patients in FLU 002 and FLU 003 with A(H1N1)pdm09 Virus Infection: a Pooled Analysis

Table 6 summarizes the association of baseline characteristics with disease progression in pooled analyses of data for FLU 002 and FLU 003 patients. The same baseline characteristics considered in the cross-sectional comparisons in Table 5 are summarized. In the unadjusted analysis, in addition to enrollment in the ICU, older age (median 48 years vs. 35 years), longer duration of symptoms (≥6 days versus <4 days), diabetes, history of CVD, renal or liver disease, and immunosuppression were significantly associated with disease progression. In multivariable analysis, enrollment in the ICU (OR 12.1, 95% CI 5.6 to 26.4; p<0.001), age (OR = 1.22 per 10 years older, 95% CI: 1.02 to 1.45; p = 0.03), duration of symptoms (≥6 days versus <4 days, OR 2.66, 95% CI 1.36 to 5.20; p = 0.004), and immunosuppression (OR 2.20, 95% CI 1.17 to 4.13; p = 0.01) were associated with disease progression.

**Table 6:** Baseline characteristics associated with disease progression: FLU002 and FLU003 pooled.

		Disease Progression		Unadjusted			Adjusted		
		Yes	No						
Characteristic		n = 109	N = 833	OR*	*95% C.I.*	P-value	OR**	*95% C.I.*	P-value
Race	Asian - %	18.3	22.9	0.78	*0.47, 1.31*	.34	1.37	*0.72, 2.62*	.34
	Black - %	7.3	4.7	1.53	*0.69, 3.38*	.30	2.21	*0.83, 5.84*	.11
	White/other - %	74.3	73.1	ref			ref		
Other demographics	Age - median (IQR)	48 (32, 57)	35 (26, 48)	1.40	*1.23, 1.59*	<.001	1.22	*1.02, 1.45*	.03
	Female - %	49.5	51.6	0.92	*0.62, 1.37*	.68	1.35	*0.84, 2.18*	.21
BMI (kg/m^2^)***	BMI<30 - %	78.0	80.3	Ref			ref		
	BMI 30-39.9 - %	18.7	16.6	1.16	*0.66, 2.03*	.61	0.83	*0.42, 1.66*	.60
	BMI≥40 - %	3.3	3.1	1.09	*0.32, 3.71*	.89	0.35	*0.09, 1.38*	.13
Onset to enrollment****	0-3 days - %	28.3	62.8	Ref			ref		
	4-5 days - %	13.2	16.2	1.81	*0.93, 3.50*	.08	1.30	*0.60, 2.78*	.51
	6+ days - %	58.5	21.0	6.16	*3.86, 9.85*	<.001	2.66	*1.36, 5.20*	.004
Season of enrollment	Oct 2009-Sep 2010 - %	62.4	67.9	0.78	*0.52, 1.18*	.25	1.36	*0.82, 2.26*	.23
	Oct 2010-Dec 2012 - %	37.6	32.1	Ref			ref		
Medical History	Smoker - %	20.4	24.5	0.79	*0.48, 1.31*	.36	0.82	*0.45, 1.47*	.50
	Asthma/COPD - %	11.9	14.5	0.80	*0.43, 1.47*	.47	0.53	*0.26, 1.06*	.07
	Diabetes - %	12.8	4.3	3.26	*1.70, 6.27*	<.001	1.33	*0.58, 3.03*	.50
	CVD/liver/renal - %	18.3	7.8	2.66	*1.54, 4.59*	<.001	1.16	*0.57, 2.38*	.68
	HIV/other immune - %	20.2	10.0	2.29	*1.36, 3.84*	.002	2.20	*1.17, 4.13*	.01
Status at enrollment	Outpatient - %	26.6	65.2	Ref			ref		
	General Ward - %	33.9	30.3	2.75	*1.65, 4.57*	<.001	1.62	*0.81, 3.24*	.17
	ICU - %	39.4	4.6	21.2	*11.9, 37.6*	<.001	12.1	*5.58, 26.4*	<.001

*Univariate odds ratio for disease progression. OR for age is for 10 years older.

**Multivariate model using imputed data with adjustment for all variables listed.

***P-value for linear trend from multivariate model = 0.16

****P-value for linear trend from multivariate model = 0.10

An analysis was performed for female patients aged ≤45 years with A(H1N1)pdm09 virus infection to investigate whether pregnancy was associated with an increased risk of disease progression. For this cohort of 336 women, among whom 29 developed disease progression, the unadjusted OR for disease progression associated with pregnancy was 4.09 (95% CI: 1.57 to 10.6; p = 0.004). With covariate adjustment, this OR was reduced and no longer significantly greater than one (OR = 1.61, 95% CI:0.42 to 6.19).

Separate analyses were carried out for patients in each study (data not shown). With few exceptions, the multivariable analyses for each study were consistent with the pooled results. In both studies, there was an increased risk of progression associated with symptoms for 6 or more versus <4 days (ORs 2.54 and 2.85 for FLU 002 and FLU 003) and immunosuppression (ORs 4.04 and 1.99). Older age was not associated with progression in FLU 002 (OR = 0.95; p = 0.80) and was associated with an increased risk of progression in FLU 003 (OR = 1.27; p = 0.02); however, the difference in the ORs was not significant (p = 0.76). Asthma or COPD was associated with a non-significant increased risk of progression in outpatients (OR = 2.22; p = 0.21) and a significant reduced risk of progression in hospitalized patients (OR = 0.35; p = 0.01) (p = 0.005 for difference in ORs). Among women aged ≤45 years, pregnancy was associated with an increased risk of progression in FLU 002 (OR = 30.1; p = 0.015) and was not associated with disease progression in FLU 003 (OR = 0.88; p = 0.89) (p = 0.07 for difference in ORs). In outpatients, there was an increased risk of progression for those enrolled during the first year (OR = 12.3; p = 0.02); this was not evident for inpatients (OR = 0.83; p = 0.57) (p = 0.06 for differences in ORs). The associations of other baseline factors considered with disease progression did not differ for FLU 002 and FLU 003 patients.

We also examined predictors of mortality during the 60-day follow-up in patients enrolled in FLU 003 (Table 7). In univariable analyses in addition to enrollment in the ICU, Asian race, duration of symptoms ≥6 days, and a history of diabetes were associated with an increased risk of death. In multivariable analyses, Asian race (p = 0.01) and duration of symptoms (p = 0.03) remained significant predictors. There was also evidence of a higher risk of death for those with immunosuppression (p = 0.03) and for those enrolled in the initial calendar period of enrollment (p = 0.01).

**Table 7:** Baseline characteristics associated with death in FLU003.

		Died		Unadjusted			Adjusted		
		Yes	No						
	Characteristic	n = 32	n = 334	OR*	*95% C.I.*	P-value	OR**	*95% C.I.*	P-value
Race	Asian - %	25.0	9.6	3.27	*1.35, 7.95*	.009	5.18	*1.48, 18.2*	.01
	Black - %	6.3	4.2	1.87	*0.40, 8.76*	.43	1.82	*0.20, 16.7*	.60
	White/other - %	68.8	86.2	ref			ref		
Other demographics	Age - median (IQR)	52 (42, 62)	46 (33, 59)	1.22	*0.97, 1.53*	.10	1.31	*0.95, 1.80*	.10
	Female - %	43.8	50.9	0.75	*0.36, 1.56*	.44	1.69	*0.65, 4.44*	.28
BMI (kg.m^2^)***	BMI<30 - %	88.0	73.4	ref			ref		
	BMI 30-39.9 - %	8.0	21.2	0.32	*0.07, 1.38*	.12	0.29	*0.06, 1.49*	.14
	BMI≥40 - %	4.0	5.4	0.61	*0.08, 4.82*	.64	0.45	*0.05, 4.14*	.48
Onset to enrollment****	0–3 days - %	6.5	24.2	ref			ref		
	4–5 days - %	16.1	23.0	2.63	*0.50, 14.0*	.26	2.77	*0.45, 16.9*	.27
	6+ days - %	77.4	52.8	5.51	*1.27, 23.9*	.02	6.20	*1.17, 32.8*	.03
Season of enrollment	Oct 2009-Sep 2010 - %	65.6	54.8	1.58	*0.74, 3.37*	.24	3.80	*1.38, 10.5*	.010
	Oct 2010-Dec 2012 - %	34.4	45.2	ref			ref		
Medical History	Smoker - %	21.4	30.0	0.64	*0.25, 1.61*	.34	1.03	*0.32, 3.38*	.96
	Asthma/COPD - %	12.5	27.8	0.37	*0.13, 1.08*	.07	0.49	*0.13, 1.79*	.28
	Diabetes - %	28.1	8.7	4.12	*1.74, 9.72*	.001	3.33	*0.97, 11.4*	.06
	CVD/liver/renal - %	31.3	18.9	1.96	*0.88, 4.33*	.10	1.15	*0.37, 3.61*	.81
	HIV/other immune - %	25.0	12.6	2.32	*0.98, 5.49*	.06	3.55	*1.10, 11.5*	.03
Status at enrollment	General ward - %	34.4	82.6	ref			ref		
	ICU - %	65.6	17.4	9.08	*4.15, 19.9*	<.001	8.97	*3.38, 23.8*	<.001

*Univariate odds ratio for death. OR for age is for 10 years older.

**Multivariate model using imputed data with adjustment for all variables listed.

***P-value for linear trend from multivariate model = 0.07

****P-value for linear trend from multivariate model = 0.35

## Discussion

In two international cohort studies of patients with A(H1N1)pdm09 virus infection, one in outpatients and the other in hospitalized patients, we estimated the risks of disease progression using several clinical outcomes. These estimates of disease progression, together with factors that influenced the risk of progression are useful considerations in designing studies aimed at the prevention and treatment of influenza infection, and planning for future epidemics. Many of the clinical outcomes we assessed have been considered in guidance from the Food and Drug Administration and were discussed at an NIH workshop [9], [10].

We found that 5% of patients seeking outpatient care required hospitalization within 14 days; almost one-half of the patients requiring hospitalization were admitted on the same day that they sought outpatient care. At 14 days, 18% of outpatients still had influenza symptoms. Other studies have also indicated that symptoms of influenza can last for many days. A prospective study conducted in the UK of 186 patients that had confirmed A(H1N1)pdm09 virus infection reported that the average duration of symptoms was 8.8 days (range 1–28 days), the average time off from work was 7.3 days (range 1–28 days), and the overall quality adjusted life days lost was 2.92 (range 0–9.84, median 2.18) [11].

In FLU 003, 13% of patients enrolled in the general ward and 53% of patients enrolled in the ICU had experienced disease progression by 60 days; mortality at 60 days was 4% and 27% for those enrolled in the general ward and ICU, respectively. At 60 days of follow-up among 288 surviving patients who were not in the hospital, 14.7% of patients (95% CI: 10.7 to 19.3%) indicated that symptoms had not resolved. There are a few other studies for which comparable results were reported, some such as reports on surveillance systems did not have a follow-up period and reported on deaths during hospitalization. In a World Health Organization study, Van Kerkhove, et al. reported on surveillance from Ministries of Health or National Public Health Institutes of 19 countries or administrative regions that encompassed 70,000 laboratory-confirmed A(H1N1)pdm09 hospitalized patients during April 2009-January 1, 2010. There were 9,700 (13.9%) patients admitted to the ICU and 2,500 (3.6%) deaths [6]. Active surveillance for laboratory-confirmed A(H1N1)pdm09 virus infection in ten U.S. states during April 2009-April 2010 found that 4% of 5238 hospitalized adults died during the hospitalization [12]. A review by Cheng using 18 published reports found that the case fatality proportion for hospitalized patients with laboratory-confirmed A(H1N1)pdm09 infection varied by region (Asia, Europe, Oceania, South America and North America) and ranged from 1.6% (Asia) to 6.9% (North America) [13]. In FLU 003, the majority of deaths (27 of 32) occurred in the hospital where the patient was enrolled. The 60-day mortality we observed among patients who were enrolled in the ICU (27%) is similar to reports by Rice and Brun-Buisson [14], [15]. Rice reported a 60-day mortality of 23% for 683 patients with confirmed or probable A(H1N1)pdm09 virus infection who were enrolled in ICUs in the United States. Brun-Buisson reported a study of 208 A(H1N1)pdm09 virus-infected patients in France with acute respiratory distress syndrome: 49 (24%) had died by 60 days following the initiation of mechanical ventilation. Kumar followed patients for 90 days and reported that among 168 critically ill patients (including 50 children) in Canada with A(H1N1)pdm09 virus infection, 29 (17.3%) patients died, including 4 children; 18 (10.7%) patients died within 14 days and 24 (14.3%) died within 28 days of critical illness onset [16]. The ANZIC Influenza Investigators reported on 722 patients with confirmed A(H1N1)pdm09 admitted to an ICU in Australia and New Zealand during June through August, 2009. The median duration of ICU stay was 7 days and 16.9% patients died in the hospital [17].

One of the notable observations associated with A(H1N1)pdm09 virus-infected patients has been that younger adult populations were affected more frequently than what is usually observed for seasonal influenza [6], [13], [16]–[23]. The median age of outpatients and inpatients in our two cohorts were 30 and 48 years, respectively. For both cohorts, the median age significantly increased after the first year. This is consistent with other reports [24], [25].

Our data suggest that morbidity and mortality during the initial season of enrollment was greater than in subsequent calendar periods after adjustment for the age difference. Consistent with this, using surveillance systems in Canada, Helferty reported a decline in admissions in the second wave of the epidemic [24]. Interestingly, a study from Spain, reported by Martin-Loeches, found a higher mortality during the post-pandemic period compared to the pandemic period; however, their analysis did not take into account the older age of patients in the post-pandemic period [25].

Our analyses also identify potential problems interpreting results from cross-sectional studies comparing outpatients and inpatients. For example, hospitalized patients were more likely to have greater BMI than outpatients; however, BMI was not associated with a risk of progression in the cohort analyses. The finding from the cross-sectional analyses may reflect the population of people that are hospitalized rather than be predictors of severe influenza. Similarly, women of child-bearing age who were pregnant were more likely to be enrolled in FLU 003 and were more likely to be hospitalized if enrolled in FLU 002. These data may reflect a reduced threshold for hospitalizing pregnant women with influenza infection because of concern about the development of disease progression. Similar findings were noted for patients with asthma or COPD. Cross-sectional differences and the apparent different associations with progression in FLU 002 and FLU 003 likely reflect a propensity for hospitalizing patients with these conditions when they develop ILI.

Longer duration of symptoms and immunosuppression were associated with an increased risk of disease progression in our study. In a previous report, we also found that markers of inflammation and coagulation were associated with an increased risk of progression [26]. Other reports have found a number of factors associated with severity of disease that include underlying chronic medical conditions, immunosuppression (including HIV if advanced immunosuppression), neurological disease, morbid obesity and pregnancy [12], [14], [18]–[21], [23]–[25], [27]–[38]. Additionally, longer duration between onset of symptoms and hospitalization has been associated with an increased risk of death or severe outcome [21], [28]

In FLU 003, the median number of days from symptom onset to enrollment was 5 days for those enrolled on the general ward and 10 days for those enrolled in an ICU. This delay in enrollment for those with severe disease is relevant for the study of new treatments as was pointed out in a recent clinical trial in Southeast Asia [39]. Approaches to expedite enrollment are important to consider when planning such studies. The finding of hospital-acquired infections emphasizes the need for influenza surveillance in the hospital setting.

Bacterial co-infections, particularly causing pneumonia, have been associated with increased severity of A(H1N1)pdm09 virus infection in hospitalized patients [14], [28]. Bacterial pneumonia was a complication found in 29% of FLU 003 participants at enrollment. Patients with influenza are thought to be at higher risk for secondary bacterial infection and pneumonia because of the cytopathic effects of viral replication in cells as well as dysregulated changes in host cytokine production that may diminish both the ability of the immune system to clear bacteria and to achieve appropriate modulation of the inflammatory cascade [40], [41]. We assessed the prevalence of viral and bacterial co-pathogens in a sample of 333 patients and did not find any significant differences in prevalence or outcomes between FLU 002 and FLU 003 patients. In a cross-sectional study of 199 patients from Argentina with A(H1N1)pdm09 virus infection, upper respiratory swabs were tested for a variety of bacterial and viral potential pathogens. In that study *S. pneumoniae* was associated with increased disease severity (it was detected among 25.0% of patients seen at ambulatory clinics and 56.4% of patients who were hospitalized or died) [42].

Approximately 66% of patients reported taking neuraminidase inhibitors (NAI) in the 14 days prior to enrollment. Of those taking antivirals, less than half started these medications within three days of illness onset. A recent meta-analysis of hospitalized patients found a decreased mortality associated with early treatment (within 48 hours of symptom onset) versus late treatment or no treatment [43]. The authors of this meta-analysis point out that sicker patients are more likely to receive antivirals and patients with milder disease may not be treated, highlighting potential confounders and limitations of observational studies.

A particular strength of our studies is that they are cohort studies with well-defined follow-up periods for estimating disease progression rates. Notably, a high proportion of enrolled patients were available for follow-up evaluation (97% for FLU 002 and 94% for FLU 003). The cohorts include patients from 17 countries, incorporating a diverse population including varied ethnicities and economies. Enrollment over a 3-year period enabled evaluation in the time period after A(H1N1)pdm09 virus emerged in 2009. Multiple clinical outcomes were assessed and described after different follow-up intervals. These data should be useful for planning intervention trials.

Of note, Ortiz and colleagues raised the concern that there is a lack of clinical studies in the setting of a public health emergency [such as the A(H1N1)pdm09 pandemic] to inform clinical care, particularly in low-resource settings [44].

By utilizing an already existing clinical study infrastructure through the INSIGHT network, we were able to rapidly develop a system for studying the emergence of a novel influenza A virus and clinical outcomes of infection in an international setting. We have maintained this system to continue observational cohort studies to assess clinical outcomes of seasonal influenza across diverse geographic areas and patient populations, and to serve as a platform for treatment studies. Further, the INSIGHT FLU network is currently being adapted to include other emerging respiratory viruses of global public health importance [e.g. MERS-CoV, avian influenza A(H7N9) virus].

Our studies have a number of limitations including the relatively small number of disease progression outcomes in the outpatient cohort, thereby limiting their power. A recent meta-analysis aimed at evaluating risk factors for severe outcomes in seasonal and pandemic influenza found that the lack of power is an issue for many studies [45]. At least a theoretical limitation is that there may be possible misclassification in FLU 003 because of potentially false positive RT-PCR results, particularly those with a positive local laboratory result and a negative central laboratory result. However, the false positive rate with commercial RT-PCR assays is generally quite low. Rather, because some of these individuals who had a positive local RT-PCR were enrolled more than ten days after the onset of symptoms, a time at which they may no longer be shedding influenza virus, the potential for misclassification would have been greater if they had been excluded.

In summary, our findings highlight the high frequency of disease progression associated with A(H1N1)pdm09 virus infection on a global basis, particularly in patients requiring hospital admission, while also highlighting the potential hazards of cross-sectional comparisons according to level of severity. Observational studies such as FLU 002 and FLU 003 that employ specified periods of clinical follow-up are absolutely critical in properly assessing disease progression and associated risk factors. Our experience will be useful in planning additional observational studies of emerging novel influenza A viruses and novel emerging respiratory viruses, and the data from FLU 002 and FLU 003 will help inform the design of interventional studies of new antiviral medications and other strategies for the treatment and prevention of influenza infection.

## Supporting Information

Table S1 **FLU 002: Local laboratory PCR vs central laboratory PCR results.** Patients enrolled through 31 Dec 2012 with results for both.
(DOC)

Table S2 **FLU 003: Local laboratory PCR vs central laboratory PCR results.** Patients enrolled through 31 Dec 2012 with results for both.
(DOC)

Appendix S1 **FLU 002 and FLU 003 participating clinical sites for which local institutional review boards or institutional ethics committees approved the FLU 002 and/or FLU 003 protocols.**
(DOC)

Appendix S2 **Comparison of local and central RT-PCR results for patients in FLU 002 and FLU 003.**
(DOC)
