# Supplementary material for: Outcomes of Influenza A(H1N1)pdm09 Virus Infection: Results from Two International Cohort Studies
Source: PLoS One. 2014 Jul 8;9(7):e101785. doi: 10.1371/journal.pone.0101785 (PMC4086938; doi:10.1371/journal.pone.0101785)
Supplement: Table S1 — FLU 002: Local laboratory PCR vs central laboratory PCR results. Patients enrolled through 31 Dec 2012 with results for both. (DOC) [file pone.0101785.s001.doc]

**Table S1. FLU 002: Local Laboratory PCR vs Central Laboratory PCR Results. Patients Enrolled through 31 Dec 2012 with Results for Both**

|  | **Central PCR** | | | | | |
| --- | --- | --- | --- | --- | --- | --- |
| **Local** | **A(H1N1)pdm09** | **A/H3N2** | **Influenza B** | **A Neg** | **A/B Neg** | **Total** |
| **PCR** | **N (Pct.)** | **N (Pct.)** | **N (Pct.)** | **N (Pct.)** | **N (Pct.)** | **N (Pct.)** |
| A(H1N1)pdm09* | 374 (81%) | 2 (1%) | 1 (1%) | 22 (6%) | 8 (1%) | 407 (22%) |
| Influenza A** | 31 (7%) | 208 (62%) | 3 (3%) | 10 (3%) | 22 (4%) | 274 (15%) |
| A/H3N2 | 0 (0%) | 14 (4%) | 0 (0%) | 0 (0%) | 0 (0%) | 14 (1%) |
| Influenza B | 2 (0%) | 1 (0%) | 14 (15%) | 0 (0%) | 2 (0%) | 19 (1%) |
| A Neg | 46 (10%) | 78 (23%) | 16 (17%) | 317 (91%) | 395 (64%) | 852 (46%) |
| A/B Neg | 6 (1%) | 30 (9%) | 61 (64%) | 0 (0%) | 190 (31%) | 287 (15%) |
| Total | 459 (100%) | 333 (100%) | 95 (100%) | 349 (100%) | 617 (100%) | 1853 (100%) |

* Influenza A positive results imputed as A(H1N1)pdm09 for earliest versions of the form.
** Influenza A, subtype unknown.
