# Supplementary material for: Outcomes of Influenza A(H1N1)pdm09 Virus Infection: Results from Two International Cohort Studies
Source: PLoS One. 2014 Jul 8;9(7):e101785. doi: 10.1371/journal.pone.0101785 (PMC4086938; doi:10.1371/journal.pone.0101785)
Supplement: Table S2 — FLU 003: Local laboratory PCR vs central laboratory PCR results. Patients enrolled through 31 Dec 2012 with results for both. (DOC) [file pone.0101785.s002.doc]

**Table S2. FLU 003: Local Laboratory PCR vs Central Laboratory PCR Results. Patients Enrolled through 31 Dec 2012 with Results for Both**

|  | **Central PCR** | | | | | |
| --- | --- | --- | --- | --- | --- | --- |
| **Local** | **A(H1N1)pdm09** | **A/H3N2** | **Influenza B** | **A Neg** | **A/B Neg** | **Total** |
| **PCR** | **N (Pct.)** | **N (Pct.)** | **N (Pct.)** | **N (Pct.)** | **N (Pct.)** | **N (Pct.)** |
| A(H1N1)pdm09* | 250 (88%) | 1 (1%) | 0 (0%) | 45 (38%) | 53 (27%) | 349 (50%) |
| Influenza A** | 14 (5%) | 43 (59%) | 1 (5%) | 0 (0%) | 36 (18%) | 94 (14%) |
| A/H3N2 | 0 (0%) | 14 (19%) | 0 (0%) | 0 (0%) | 0 (0%) | 14 (2%) |
| Influenza B | 0 (0%) | 0 (0%) | 4 (20%) | 0 (0%) | 2 (1%) | 6 (1%) |
| A Neg | 19 (7%) | 12 (16%) | 13 (65%) | 73 (62%) | 60 (30%) | 177 (26%) |
| A/B Neg | 1 (0%) | 3 (4%) | 2 (10%) | 0 (0%) | 47 (24%) | 53 (8%) |
| Total | 284 (100%) | 73 (100%) | 20 (100%) | 118 (100%) | 198 (100%) | 693 (100%) |

* Influenza A positive results imputed as A(H1N1)pdm09 for earliest versions of the form.
** Influenza A, subtype unknown.
