# Supplementary material for: Outcomes of Influenza A(H1N1)pdm09 Virus Infection: Results from Two International Cohort Studies
Source: PLoS One. 2014 Jul 8;9(7):e101785. doi: 10.1371/journal.pone.0101785 (PMC4086938; doi:10.1371/journal.pone.0101785)
Supplement: Appendix S1 — FLU 002 and FLU 003 participating clinical sites for which local institutional review boards or institutional ethics committees approved the FLU 002 and/or FLU 003 protocols. (DOC) [file pone.0101785.s003.doc]

**Appendix S1. FLU 002 and FLU 003 Participating Clinical Sites for which**

**Local Institutional Review Boards or Institutional Ethics Committees Approved the FLU 002 and/or FLU 003 Protocols**

12th Respiratory and TB Medical Dept, Athens Hosp for Diseases of the Chest "Sotiria Hospital", Athens, Greece

1st Respiratory Medicine Dept, Athens Hospital for Diseases of the Chest "Sotiria Hospital", Athens, Greece

2nd Internal Medicine Department, Sismanoglio Athens General Hospital, Athens, Greece

3rd Pulmonary Department, Sismanoglio Athens General Hospital, Athens, Greece

Aalborg Hospital, Aalborg, Denmark

Arhus Universitetshospital, Skejby, Aarhus, Denmark

Attikon University General Hospital, Athens, Greece

Baystate Infectious Diseases Clinical Research, Springfield, MA, United States

Brigham and Women's Hospital, Boston, MA, United States

Brighton and Sussex University Hospitals NHS Trust, Brighton, United Kingdom

CAICI (Instituto Centralizado de Assistencia e Investigación Clínica Integral), Rosario, Argentina

CEMIC, Buenos Aires, Argentina

CHIP, Copenhagen, Denmark

Cairns Sexual Health Service, Cairns North, Australia

Cambridge Health Alliance, Cambridge, MA, United States

Centre Hospitalier Universitaire St. Pierre (C.H.U. St. Pierre), Brussels, Belgium

Chulalongkorn University Hospital, Bangkok, Thailand

Churchill Hospital, Headington, United Kingdom

Cooper University Hospital, Camden, NJ, United States

Cornell CRS, New York, NY, United States

David Geffen School of Medicine at UCLA, Los Angeles, CA, United States

Duke University, Durham, NC, United States

EMC Instytut Medyczny SA, Wroclaw, Poland

FUNCEI, Buenos Aires, Argentina

Fundación Arriarán, Santiago, Chile

George Washington Medical Faculty Associates, Washington, DC, United States

Hennepin County Medical Center, Minneapolis, MN, United States

Hippokration University General Hospital of Athens, Athens, Greece

Holdsworth House Medical Practice, Darlinghurst, Australia

Hospital Carlos III, Madrid, Spain

Hospital Clínico San Carlos, Madrid, Spain

Hospital General de Agudos JM Ramos Mejia, Buenos Aires, Argentina

Hospital Interzonal General de Agudos Dr. Diego Paroissien, La Matanza, Argentina

Hospital Italiano de Buenos Aires, Buenos Aires, Argentina

Hospital La Paz, Madrid, Spain

Hospital Nacional Arzobispo Loayza, Lima, Peru

Hospital Nacional Guillermo Almenara Irigoyen, Lima, Peru

Hospital Privado Centro Médico de Córdoba, Cordoba, Argentina

Hospital Profesor Bernardo Houssay, Vicente Lopez, Argentina

Hospital Rawson, Cordoba, Argentina

Hospital Universitari Mutua Terrassa, Terrassa, Spain

Hospital Universitario Gregorio Marañón, Madrid, Spain

Hospital Universitario Príncipe de Asturias, Alcala de Henares, Spain

Hospital del Mar, Barcelona, Spain

Hvidovre University Hospital, Department of Infectious Diseases, Hvidovre, Denmark

Ifi - Studien und Projekte GmbH, Hamburg, Germany

Infectious Diseases Associates NW FL, PA, Pensacola, FL, United States

Institute of Tropical Medicine, Antwerp, Belgium

Interchange General Practice, Canberra, Australia

Johann Wolfgang Goethe - University Hospital, Infektionsambulanz CRS, Frankfurt, Germany

Khon Kaen University, Srinagarind Hospital, Khon Kaen, Thailand

Klinik I für Innere Medizin der Universität zu Köln, Studienbüro für Infektiologie u. HIV, Cologne, Germany

Klinikum der Universität München, Munich, Germany

Mayo Clinic, Rochester, MN, United States

Medizinische Universitätsklinik - Bonn, Immunologische Ambulanz CRS, Bonn, Germany

Miami Valley Hospital, Dayton, OH, United States

Montefiore Medical Center, Bronx, NY, United States

New Jersey Medical School Adult Clinical Research Center, Newark, NJ, United States

Northside Clinic, North Fitzroy, Australia

Odense University Hospital, Odense, Denmark

Oslo University Hospital, Ulleval, Oslo, Norway

Pontificia Universidad Catolica de Chile, Santiago, Chile

Practimed Medisch Centrum Tessenderlo, Tessenderlo, Belgium

Prahran Market Clinic, Melbourne, Australia

Queen Elizabeth Hospital, Kowloon, China

Rigshospitalet, Infektionsmedicinsk ambulatorium 8622, Copenhagen, Denmark

Ruhr-University, Bochum, Germany

SUNY Downstate Medical Center, Brooklyn, NY, United States

Sheffield Teaching Hospital NHS Foundation Trust, Sheffield, United Kingdom

St. Vincent's Hospital, Darlinghurst, Australia

Taylor Square Private Clinic, Darlinghurst, Australia

The Alfred Hospital, Melbourne, Australia

The James Cook University Hospital, Middlesbrough, United Kingdom

UCSD Antiviral Research Center, San Diego, CA, United States

University Hospital Charite, Berlin, Germany

University Vienna General Hospital, Vienna, Austria

Universitätsklinikum Würzburg, Medizinische Klinik und Poliklinik II, Schwerpunkt Infektiologie CRS, Wuerzburg, Germany

Washington DC VA Medical Center, Washington, DC, United States

West Tallinn Central Hospital Infectious Diseases, Tallinn, Estonia

Westmead Hospital, Westmead, Australia

Wojewodzki Szpital Zakazny, Warsaw, Poland
