# Supplementary material for: Outcomes of Influenza A(H1N1)pdm09 Virus Infection: Results from Two International Cohort Studies
Source: PLoS One. 2014 Jul 8;9(7):e101785. doi: 10.1371/journal.pone.0101785 (PMC4086938; doi:10.1371/journal.pone.0101785)
Supplement: Appendix S2 — Comparison of local and central RT-PCR results for patients in FLU 002 and FLU 003. (DOC) [file pone.0101785.s004.doc]

**Appendix S2. Comparison of Local and Central RT-PCR Results for Patients in FLU 002 and FLU 003**

Tables S1 and S2 compare local and central RT-PCR results for patients in FLU 002 and FLU 003, respectively. In FLU 002, swabs taken for central and local laboratory testing were obtained at the same time. In contrast, for the hospitalized patients in FLU 003, local RT-PCR testing was frequently performed earlier, and the enrollment of FLU 003 patients occurred longer following the onset of ILI symptoms as compared to outpatients in FLU 002.

Among 1,853 patients in FLU 002 with central and local laboratory RT-PCR results, 52 of 459 (11.3%) patients with a central laboratory determination of A(H1N1)pdm09 virus infection were influenza A negative by local laboratory testing. Among the 407 patients with A(H1N1)pdm09 virus infection by local laboratory testing, 30 (7.4%) were influenza A negative by central laboratory testing (Table S1). For this comparison, we assumed patients initially enrolled with a positive RT-PCR test result for influenza A, but with subtype not reported, were positive for A(H1N1)pdm09. This assumption was based on the fact that initial enrollments in 2009-2010 occurred only where A(H1N1)pdm09 virus was circulating. Furthermore, during the time period of enrollment before local RT-PCR results for influenza A subtypes were recorded, 100% of 320 central results that were influenza A positive yielded A(H1N1)pdm09.

Among 693 patients enrolled in FLU 003 with local and central laboratory RT-PCR test results, 20 of 284 (7.0%) patients with central laboratory-confirmed A(H1N1)pdm09 virus infection were influenza A negative by local laboratory testing. Among the 349 patients with A(H1N1)pdm09 identified by local laboratory RT-PCR testing, or who were enrolled before test results for influenza A subtypes were recorded, 98 (28.0%) were influenza A negative by central laboratory testing (Table S2). For this summary, like the one for FLU 002, we assumed patients initially enrolled with a local laboratory positive RT-PCR test result for influenza A were positive for A(H1N1)pdm09. This assumption was based on the fact that initial enrollments only occurred where A(H1N1)pdm09 virus was circulating and during the time period of enrollment before local laboratory RT-PCR results for influenza A subtypes were recorded, all 147 patients with central results that were influenza A were positive for A(H1N1)pdm09.

For FLU 003, across the entire time period covered by this report, the percentage with a local laboratory RT-PCR test result positive for A(H1N1)pdm09 and a central laboratory result negative for influenza A negative was much higher than in FLU 002 (28.0% versus 7.4%). For the 98 patients with positive local laboratory test results for A(H1N1)pdm09 and negative for influenza A at a central laboratory, the swab obtained for central laboratory testing was obtained a median of 4 days (IQR: 2 to 7) after the swab obtained for local laboratory testing; 7 of the 98 patients (7.1%) had swabs for local and central laboratory testing obtained on the same day. By comparison, for the 250 patients who were positive for A(H1N1)pdm09 at both a local and central laboratory, this time difference was 2 days (IQR: 1 to 3); for 45 of the 250 patients (18%), the swabs were obtained on the same day.

We concluded from these results that some patients in FLU 003 were incorrectly classified centrally as negative for A(H1N1)pdm09 because swabs were obtained for central laboratory testing after swabs were collected for local laboratory testing, and at that time they were likely no longer shedding detectable A(H1N1)pdm09 virus. Overall the median time between collection of the local and central swabs was 2 (IQR: 1 to 4) days. We also concluded from these analyses that for the FLU 002 cohort, the lower percent of discordant local and central laboratory results than in FLU 003 was likely due to the samples for local and central laboratory testing being obtained at the same time and closer to the onset of symptoms than for patients in FLU 003. For FLU 002, the discordance could be attributable to measurement error associated with obtaining the samples and carrying out the tests.

As described in Methods, based on these results, the FLU 002 cohort was comprised of patients enrolled with A(H1N1)pdm09 by RT-PCR by a central laboratory. Local laboratory results were not used to define this cohort.

The FLU 003 hospitalized cohort includes: 1) patients with central laboratory -confirmed A(H1N1)pdm09; 2) patients that tested A(H1N1)pdm09 positive by local laboratory testing who tested influenza A negative by RT-PCR at a central laboratory; and 3) patients who tested influenza A positive by RT-PCR at a local laboratory and who tested influenza A negative by RT-PCR at a central laboratory during the initial 6 months of enrollment when A(H1N1)pdm09 virus was prevalent and the results of local laboratory RT-PCR testing did not record the influenza A virus subtype.
